# Unlocking Hopeaphenol: A Potent Ally Against Cardiac Hypertrophy via AMPK Activation

**DOI:** 10.3390/nu17183025

**Published:** 2025-09-22

**Authors:** Jinhong Chen, Mengyuan Wang, Zhongzheng Zhang, Chongkai Fang, Haowen Zhuang, Jiaqi Zhao, Tianyu Wang, Junyan Wang, Chun Li, Chunping Fang

**Affiliations:** 1 School of Basic Medical Sciences, Guangzhou University of Chinese Medicine, Guangzhou 510006, China; 18002630878@163.com (J.C.); wmy8762@163.com (M.W.); 2 School of Pharmaceutical Sciences, Guangzhou University of Chinese Medicine, Guangzhou 510006, China; 18381497949@163.com (Z.Z.); haowen_zhuang@163.com (H.Z.); 13140096664@163.com (J.Z.); 13091428177@163.com (T.W.); junyan_wang@163.com (J.W.); 3 Science and Technology Innovation Center, Guangzhou University of Chinese Medicine, Guangzhou 510405, China; f.chongkai@gzucm.edu.cn

**Keywords:** hopeaphenol, AMPK signaling pathway, cardiac hypertrophy, mitochondrial function, heart failure

## Abstract

**Background:** Abnormal mitochondrial energy metabolism is a key factor in the development and progression of cardiac hypertrophy. Hopeaphenol (HP), a tetramer of the natural polyphenol resveratrol, exhibits higher biological activity than resveratrol, but its specific role in cardiac hypertrophy and underlying mechanisms remains unclear. **Methods:** This study explored the protective effect and mechanism of hopeaphenol on cardiac hypertrophy through in vivo and in vitro experiments. In in vivo experiments, transverse aortic constriction (TAC) was used to induce cardiac hypertrophy in mice; HE, Masson, and WGA staining were applied to observe myocardial changes, ELISA was used to detect animal serum indicators, and the Cellular Thermal Shift Assay (CETSA) was conducted to verify the interaction between hopeaphenol and AMPK. In in vitro experiments, angiotensin II (Ang II) was used to induce hypertrophy of HL-1 cardiomyocytes, and the AMPK-specific inhibitor Compound C was employed to confirm the role of the AMPK pathway. **Results:** In in vivo experiments, TAC-induced cardiac hypertrophy in mice was characterized by left ventricular cavity enlargement and decreased ejection fraction; hopeaphenol treatment significantly improved these cardiac function indices, and HE, Masson, and WGA staining confirmed that hopeaphenol could restore cardiomyocyte morphology and reduce fibrosis. ELISA results of animal serum showed that hopeaphenol could improve metabolic disorders in TAC mice. Furthermore, CETSA confirmed a direct interaction between hopeaphenol and AMPK. In in vitro experiments, hopeaphenol reduced Ang II-induced hypertrophy and apoptosis of HL-1 cardiomyocytes, enhanced mitochondrial membrane potential, and decreased reactive oxygen species (ROS) levels by activating the AMPK pathway; moreover, the AMPK-specific inhibitor Compound C blocked these effects. This suggests that hopeaphenol’s cardioprotective effect is largely mediated by AMPK activation. **Conclusions:** The protective effect of hopeaphenol on cardiac hypertrophy is highly dependent on the activation of the AMPK signaling pathway, with CETSA and molecular docking supporting direct binding between hopeaphenol and AMPK; this pathway improves mitochondrial dysfunction through AMPK, thereby alleviating heart failure caused by pressure overload. This finding identifies hopeaphenol as a potential candidate for further development in the prevention and treatment of heart failure.

## 1. Introduction

Cardiac hypertrophy represents an adaptive response of the heart to conditions of pressure or volume overload. Initially, this adaptation can preserve cardiac function; however, sustained overload results in pathological remodeling, which is characterized by myocardial fibrosis, arrhythmias, and ultimately progression to heart failure [1,2,3,4]. Disruptions in energy metabolism play a crucial role in the onset and progression of cardiac hypertrophy. A fundamental driving factor is the heart’s high energy demand, which relies on ATP production through mitochondrial oxidative phosphorylation [5]. Disorders in cardiac energy metabolism often precede the development of hypertrophy [6]. Increased cardiac load disrupts mitochondrial homeostasis, leading to excessive production of reactive oxygen species (ROS) [7]. The accumulation of ROS exacerbates disturbances in energy metabolism by activating pro-inflammatory and pro-apoptotic signaling pathways, thereby accelerating the transition from cardiac hypertrophy to heart failure [8,9,10].

AMP-activated protein kinase (AMPK), a critical regulator of energy metabolism, is activated in response to pressure overload and plays an essential role in maintaining mitochondrial homeostasis by modulating downstream target proteins, thereby exerting a significant influence on cardiac metabolism [11,12,13]. Although the natural polyphenol resveratrol is known to ameliorate oxidative stress, its clinical application is constrained by its low bioavailability and adverse effects at high doses, such as gastrointestinal discomfort and thyroid dysfunction [14,15,16,17]. Previous studies have shown that hopeaphenol, a resveratrol tetramer, exhibits cardioprotection-related activities—including anti-apoptosis and mitochondrial function regulation—and displays functional differences from other resveratrol tetramers [18]; however, its effects on cardiac hypertrophy and the underlying mechanism of AMPK modulation remain unclear.

Considering the potential of hopeaphenol in modulating mitochondrial function and the pivotal role of AMPK in energy metabolism, this study posits that hopeaphenol may alleviate pressure overload-induced cardiac hypertrophy by activating the AMPK signaling pathway, with mechanisms associated with the amelioration of mitochondrial metabolic abnormalities. This hypothesis was validated through in vivo and in vitro experiments to identify a novel therapeutic target for the prevention and treatment of heart failure. Furthermore, this investigation elucidates the structure-activity relationship of natural polyphenols, highlights the advantages of tetrameric structures over monomeric forms, and provides a theoretical framework for addressing the limitations of existing pharmacological agents, such as the dosage constraints associated with resveratrol. Consequently, this research holds significant implications for both fundamental studies and clinical applications.

## 2. Materials and Methods

### 2.1. Experimental Materials

#### 2.1.1. Experimental Animals

Male C57BL/6 mice (wild-type, specific pathogen-free [SPF]) aged 6–8 weeks (weight: 20–25 g) were used in this study. The mice were purchased from the Experimental Animal Center of Guangzhou University of Chinese Medicine. All animals were housed in the SPF-grade animal facility of Guangzhou University of Chinese Medicine under controlled conditions (temperature: 22 ± 2 °C, humidity: 50% ± 10%, 12 h light-dark cycle) with ad libitum access to standard laboratory chow and water. Environmental enrichment (nesting materials and plastic shelters) was provided in each cage for the mice. The animal study was reviewed and approved by the Ethics Committee of Guangzhou University of Chinese Medicine (Approval No. XS20240031, approved on 19 March 2024).

#### 2.1.2. Cell Line

The HL-1 mouse cardiomyocyte cell line was purchased from iCellverse and cultured in DMEM medium containing 10% fetal bovine serum (FBS) and 100 U/mL penicillin/streptomycin in a 37 °C, 5% CO_2_ incubator.

#### 2.1.3. Main Reagents, Consumables, and Instruments

Hopeaphenol (≥98%, #388582-37-4), was purchased from Sigma-Aldrich (St. Louis, MO, USA). Compound C (#S7840) and Angiotensin II (Ang II, #HY-P0108) were obtained from Selleck Chemicals (Houston, TX, USA) and MedChemExpress (MCE, Monmouth Jct., NJ, USA), respectively. The related antibodies used in this study included anti-phospho-AMPK (pAMPK, #83924-1-RR, Proteintech Group, Rosemont, IL, USA), anti-AMPK (#AB32047, Abcam, Cambridge, UK), and anti-SIRT1 (#DF6033, Affinity Biosciences, Cincinnati, OH, USA). Detection kits were sourced as follows: Superoxide Dismutase (SOD) assay kit (#BC5165, Solarbio Life Sciences, Beijing, China); Adenosine Triphosphate (ATP) assay kit (#S0026, Beyotime Biotechnology, Shanghai, China); Reactive Oxygen Species (ROS) assay kit (#S0033S, Beyotime Biotechnology, Shanghai, China); and JC-1 mitochondrial membrane potential assay kit (#C2006, Beyotime Biotechnology, Shanghai, China).Instruments included the ChemiScope S6 imager, ViiA 7 real-time PCR system, Varioskan LUX microplate reader, etc.

### 2.2. Experimental Methods

#### 2.2.1. Animal Model Construction and Grouping

Thirty-six C57BL/6 mice were randomly assigned to six experimental groups (*n* = 6 per group) using a computer-generated random number sequence. The specific grouping and treatment protocols for each group were as follows: (1) Sham group: Mice underwent thoracotomy without aortic ligation; (2) Transverse Aortic Constriction (TAC) model group: Cardiac hypertrophy was induced via TAC surgery [19]; (3) Hopeaphenol (HP) low-dose group: Mice received an intraperitoneal injection of 5 mg/kg HP after TAC surgery; (4) HP medium-dose group: Mice received an intraperitoneal injection of 10 mg/kg HP after TAC surgery; (5) HP high-dose group: Mice received an intraperitoneal injection of 20 mg/kg HP after TAC surgery; and (6) Metformin group: Mice received an intraperitoneal injection of 200 mg/kg metformin after TAC surgery.

The dosage of HP was determined based on preliminary experiments. These pre-experiments showed that doses below 5 mg/kg exerted minimal cardioprotective effects, while doses exceeding 20 mg/kg failed to enhance therapeutic efficacy and instead led to increased drug exposure. In accordance with standard TAC model protocols, 200 mg/kg metformin was used as a positive control. This dose was selected based on: (1) species-specific pharmacokinetics—rodents (especially mice) have higher metabolic rates and faster drug clearance than humans, requiring higher mg/kg doses to achieve comparable pharmacologically active exposure; (2) established efficacy in TAC models, as 200 mg/kg (intraperitoneal injection) is widely validated for demonstrating metformin’s AMPK-mediated cardioprotection [20,21]. Drug administration was initiated 4 weeks after TAC surgery and continued for an additional 4 weeks. This timing was intentionally chosen to investigate hopeaphenol’s potential for therapeutic reversal of established cardiac hypertrophy and dysfunction (rather than prevention), as: (1) clinically, patients typically seek treatment after symptom onset and disease establishment, making a reversal strategy more translationally relevant; (2) allowing hypertrophy to fully develop before intervention ensures observed improvements are attributed to direct therapeutic effects of hopeaphenol, not prevention of initial adaptive responses. The Sham group and TAC model group were not given any drugs; instead, they received intraperitoneal injections of an equal volume of normal saline.

#### 2.2.2. Inclusion and Exclusion Criteria

All male C57BL/6 mice (6–8 weeks old, 20–25 g) undergoing transverse aortic constriction (TAC) surgery were included in the study. No animals were excluded from the analysis prior to the experimental endpoint (8 weeks post-TAC, including 4 weeks of drug administration) unless specified otherwise. Mice that died during TAC surgery or within the first 48 h post-operation (*n* = 2) were classified as surgical fatalities (not related to experimental intervention) and were replaced with age- and weight-matched mice to maintain the predetermined group size (*n* = 6 per group). Data points from all surviving mice were included in the final statistical analysis, and no data were excluded post hoc, in accordance with the predefined experimental protocol.

#### 2.2.3. Hopeaphenol Solution Preparation

Hopeaphenol was dissolved in DMSO, mixed according to a ratio of DMSO:PEG-300:normal saline = 1:8:11, stored at 4 °C in the dark, and confirmed to be free of precipitation before use.

#### 2.2.4. Cardiac Function Detection

At the end of the experiment, detection was performed using a small animal ultrasound imaging system. Mice were anesthetized with isoflurane (2%, oxygen flow rate: 1.5 L/min), and hemodynamic indices such as left ventricular ejection fraction (LVEF) and left ventricular fractional shortening (LVFS) were measured.

#### 2.2.5. Tissue Staining

The left ventricular tissues were fixed, and hematoxylin and eosin (HE) staining was used to observe cardiomyocyte morphology. Masson staining was used to evaluate fibrosis, and WGA staining combined with ImageJ (Fiji distribution, version 2.15.0) was used to measure cardiomyocyte cross-sectional area (five random fields per section).

#### 2.2.6. Western Blot

Total proteins from tissues or cells were extracted and quantified using the BCA method. The proteins were then subjected to SDS-PAGE, transferred to PVDF membranes, and blocked. Following this, the membranes were incubated with primary antibodies AMPK, pAMPK, and SIRT1) and secondary antibodies. The proteins were detected using chemiluminescence, and protein expression was analyzed.

#### 2.2.7. qRT-PCR Detection

RNA was extracted and reverse-transcribed into cDNA. The SYBR Green method was used for amplification, with glyceraldehyde 3-phosphate dehydrogenase (GAPDH) as the internal reference. The relative expression levels of target genes (AMPK, SIRT1, and others) were calculated using the 2^−ΔΔCt^ method (primer sequences are shown in Table 1).

#### 2.2.8. CCK-8 Assay for Cell Viability

HL-1 cells were seeded in 96-well plates (5 × 10^4^ cells/mL), cultured for 24 h, and then treated with hopeaphenol at concentrations of 1 μM, 5 μM, 10 μM, 20 μM, and 50 μM, as well as Compound C, for 12 h, with a blank control group included. Subsequent procedures were performed according to the instructions.

#### 2.2.9. Cell Modeling, Grouping, and Administration

HL-1 cells were pretreated with hopeaphenol (1–50 μM) for 2 h, and then induced to undergo hypertrophy by 1 μM Ang II for 24 h. In inhibition groups, 10 μM Compound C was added 1 h prior to hopeaphenol treatment.

Dose rationale: HP and CC concentrations (1–50 μM) were determined via CCK-8, with 1–10 μM as a non-toxic range (no significant viability reduction), referencing stilbene use in cardiomyocyte studies (1–20 μM for anti-hypertrophy). Ang II (1 μM) [22] and Compound C (10 μM) followed standard protocols for inducing hypertrophy and inhibiting AMPK.

#### 2.2.10. Mitochondrial ROS Detection

HL-1 cells were seeded in culture plates and cultured to an appropriate density, then incubated with 20 μM DCFH-DA fluorescent probe in the dark for 30 min. After washing with PBS to remove excess probe, detection was performed by fluorescence microscopy.

#### 2.2.11. Mitochondrial Membrane Potential Detection

Mitochondrial membrane potential was detected by JC-1 staining. Treated cardiomyocytes were incubated with JC-1 working solution for 20 min, washed three times with PBS, and the intensities of red and green fluorescence were observed under a fluorescence microscope. The ratio of red to green fluorescence intensity was calculated to evaluate changes in mitochondrial membrane potential.

#### 2.2.12. Cell Thermal Shift Assay (CETSA)

Cells were incubated with 10 μM hopeaphenol for 2 h, then heated at a gradient of 37–65 °C. The residual amount of AMPK protein was detected by Western blotting.

### 2.3. Network Pharmacology Analysis

#### 2.3.1. Screening of Active Components and Targets

Hopeaphenol was obtained from the TCMSP database and screened according to the criteria of oral bioavailability (OB) ≥ 30% and drug-likeness (DL) ≥ 0.18. The results were converted to human gene names via the UniProt database.

#### 2.3.2. Acquisition of Heart Failure Target Genes

The term “Heart Failure” was employed to search the OMIM and GeneCards databases. The data retrieved from these sources was consolidated, and any duplicate entries were eliminated.

#### 2.3.3. Screening of Intersection Targets

A comparative analysis between the targets of hopeaphenol and those associated with heart failure was conducted using the EVenn (version 3.0.0) tool, resulting in the identification of potential therapeutic targets at the intersection.

#### 2.3.4. Construction of Hopeaphenol-Heart Failure Target Network

The intersection targets identified between hopeaphenol and heart failure were imported into Cytoscape version 3.9.1. In this network, node degree values were utilized to indicate the strength of the associations.

#### 2.3.5. Construction of PPI Network and Screening of Core Targets

Intersection targets were imported into the STRING database (species: Homo sapiens, confidence threshold > 0.9, with disconnected nodes excluded). Subsequently, the data were imported into Cytoscape software, where topological parameters were calculated to identify core targets.

#### 2.3.6. GO and KEGG Enrichment Analyses

Potential targets underwent enrichment analysis using the DAVID database. The results were filtered based on a predetermined *p*-value threshold and visualized accordingly.

#### 2.3.7. Molecular Docking Analysis

The structure of AMP-activated protein kinase (AMPK) was retrieved from the Protein Data Bank (PDB). For docking analysis, we selected two structures: the crystal structure of the human AMPK α2 kinase domain (PDB ID: 2H6D, the predominant cardiac catalytic isoform) and the full-length active-state AMPK heterotrimer (PDB: 6B2E). After optimizing the hopeaphenol structure, AutoDock Vina version 1.2.5 was used to calculate binding energy and assess complex stability. We note that 2H6D only includes the kinase domain (no β/γ regulatory subunits), so these results preliminarily explore catalytic site interactions; future studies will investigate allosteric modulation via β/γ subunits.

### 2.4. Statistical Analysis

Experimental data were analyzed using GraphPad Prism 9.5 software. Comparisons among multiple groups were performed by one-way analysis of variance (one-way ANOVA), and pairwise comparisons between groups were performed using the LSD or Dunnett’s method. A *p*-value is significant.

## 3. Results

### 3.1. Hopeaphenol Significantly Enhances Cardiac Function in TAC Mice

**Figure 1:**
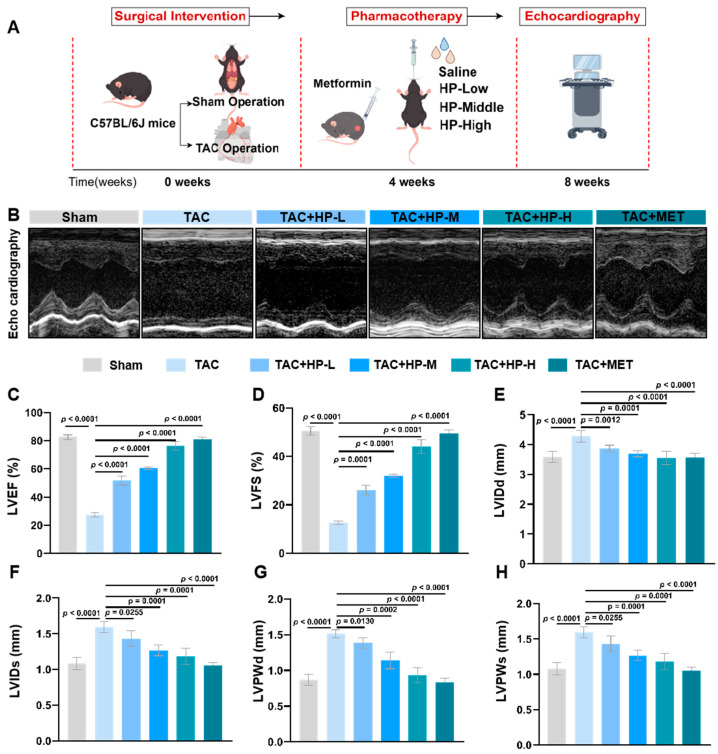
Hopeaphenol improves cardiac function in TAC-induced heart failure mice. (**A**) Mouse modeling flowchart. (**B**) Representative echocardiographic images. (**C**–**H**) Changes in LVEF, LVFS, LVIDd, LVIDs, LVPWd, and LVPWs in each group. Exact *p*-values for all post hoc comparisons are indicated on the graphs. Data are presented as mean ± SEM (*n* = 6). HP, Hopeaphenol; MET, Metformin.

Compared to the Sham group, the TAC group exhibited a marked reduction in left ventricular ejection fraction (LVEF, Figure 1C) and left ventricular fractional shortening (LVFS, Figure 1D), alongside a significant increase in left ventricular end-diastolic dimension (LVIDd, Figure 1E), left ventricular end-systolic dimension (LVIDs, Figure 1F), left ventricular posterior wall thickness at end-diastole (LVPWd, Figure 1G), and left ventricular posterior wall thickness at end-systole (LVPWs, Figure 1H). These findings confirm the successful modeling of TAC and the induction of cardiac hypertrophy and dysfunction. In contrast, the cardiac function-related indices in the hopeaphenol treatment groups demonstrated significant improvements in a dose-dependent manner, with the high-dose group exhibiting the most pronounced effects, comparable to those observed in the metformin group (Figure 1).

### 3.2. Hopeaphenol Mitigates Cardiac Hypertrophy and Fibrosis in TAC Mice

**Figure 2:**
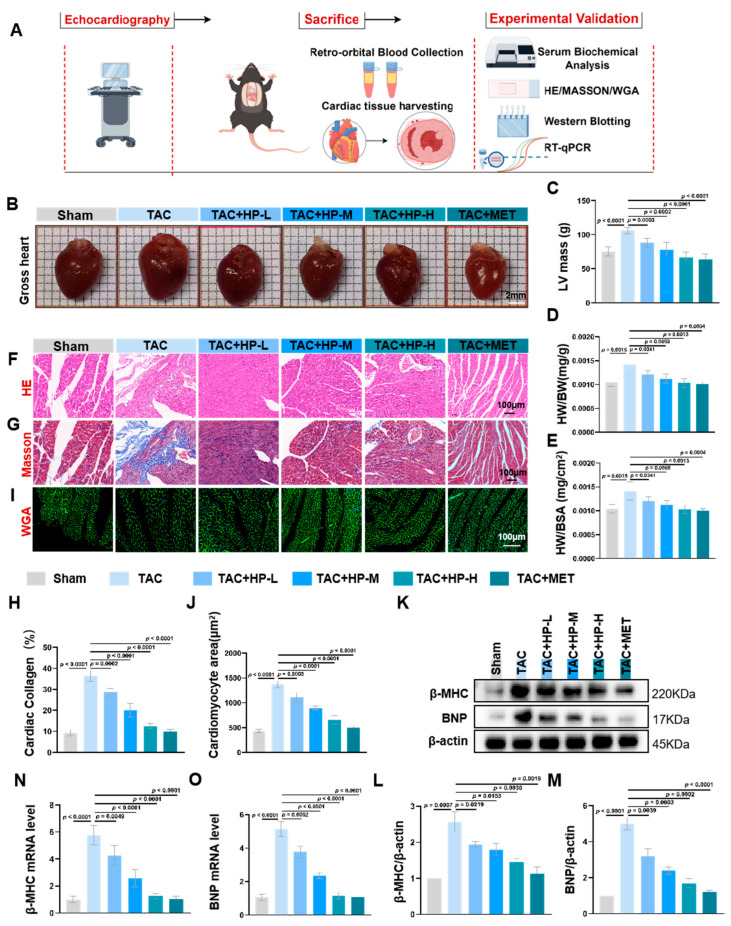
Hopeaphenol attenuates TAC-induced pathological cardiac remodeling. (**A**) Mouse modeling flowchart. (**B**) Gross heart morphology (scale bar, 2 mm). (**C**–**E**) Changes in LV mass, HW/BW, and HW/BSA in each group. (**F**–**J**) Representative H&E (**F**), Masson (**G**,**H**), and WGA (**I**,**J**) staining (scale bar, 100 μm for H&E/Masson/WGA). (**K**–**M**) Western blot bands of β-MHC and BNP (**K**) with densitometric analysis (**L**,**M**). (**N**,**O**) mRNA levels of β-MHC and BNP in myocardium. Exact *p*-values for all post hoc comparisons are indicated on the graphs. Data are presented as mean ± SEM (*n* = 6). HP, Hopeaphenol; MET, Metformin.

In comparison to the Sham group, the TAC group exhibited a significant increase in heart volume (Figure 2B), left ventricular mass (LV Mass, Figure 2C), heart weight-to-body weight ratio (Figure 2D), and heart weight-to-body surface area ratio (Figure 2E). Hematoxylin and eosin (HE) staining revealed that cardiomyocytes in the TAC group were markedly hypertrophic and exhibited morphological disarray. In contrast, the degree of cardiomyocyte hypertrophy in the hopeaphenol treatment groups was significantly diminished, with morphology appearing more regular (Figure 2F). Masson staining indicated a significant increase in collagen fiber deposition and exacerbated fibrosis within the myocardial tissue of the TAC group, whereas cardiac fibrosis was mitigated in the hopeaphenol treatment groups (Figure 2G,H). Wheat germ agglutinin (WGA) staining demonstrated a significant enlargement in the cross-sectional area of cardiomyocytes in the TAC group, with hypertrophy being alleviated in the hopeaphenol treatment groups (Figure 2I,J). Furthermore, Western blot and quantitative PCR analyses revealed that the protein and mRNA expression levels of BNP and β-MHC in the myocardial tissue of the TAC group were significantly elevated, whereas these levels were reduced in the hopeaphenol treatment group (Figure 2K–O). The aforementioned findings suggest that hopeaphenol ameliorates TAC-induced cardiac hypertrophy and fibrosis in a dose-dependent fashion, exhibiting effects comparable to those observed in the metformin treatment group (Figure 2).

### 3.3. Hopeaphenol Alleviates Oxidative Stress and Metabolic Issues

**Figure 3:**
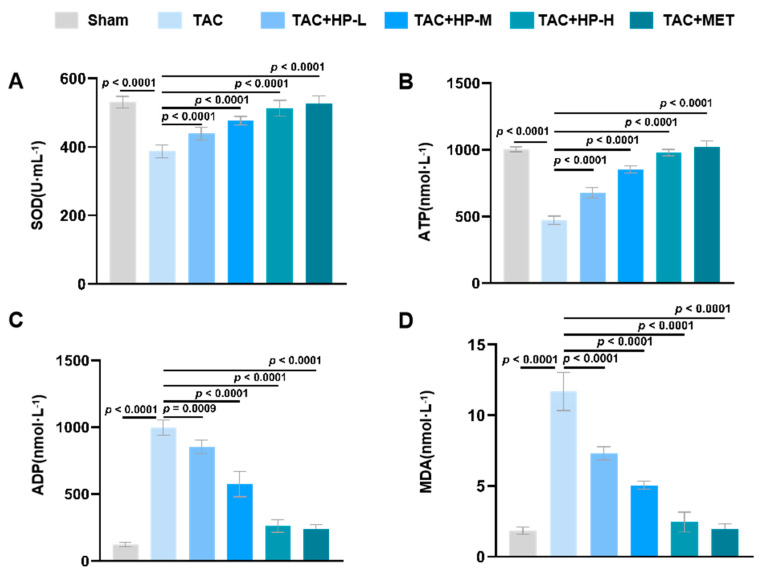
Hopeaphenol ameliorates oxidative stress and metabolic dysregulation in TAC mice. (**A**–**D**) Changes in serum SOD activity, ATP, ADP, and MDA levels in each group. Exact *p*-values for all post hoc comparisons are indicated on the graphs. Data are presented as mean ± SEM (*n* = 6). HP, Hopeaphenol; MET, Metformin.

In the TAC group, myocardial tissue showed significant oxidative stress and metabolic issues. Superoxide dismutase (SOD) activity (Figure 3A), crucial for combating reactive oxygen species (ROS), was notably reduced, indicating diminished oxidative stress defenses. Concurrently, serum adenosine triphosphate (ATP) levels (a marker of systemic energy metabolism, Figure 3B), vital for reflecting whole-body metabolic status, dropped significantly, while serum adenosine diphosphate (ADP) levels (Figure 3C) rose, pointing to a systemic metabolic disturbance in TAC mice. Additionally, malondialdehyde (MDA), a systemic oxidative stress indicator, was significantly elevated (Figure 3D). Hopeaphenol treatment reduced these issues in a dose-dependent way by boosting SOD activity, enhancing antioxidant defenses, lowering ROS levels, increasing serum ATP production, and decreasing ADP accumulation, thereby restoring energy balance. To directly assess cardiac mitochondrial function, in vitro experiments (Section 3.6) measured mitochondrial membrane potential and ROS in HL-1 cells, providing direct evidence of HP’s beneficial effects on cardiac mitochondrial function (Figure 3).

### 3.4. Hopeaphenol Exerts Cardioprotective Effects by Targeting the AMPK Pathway

**Figure 4:**
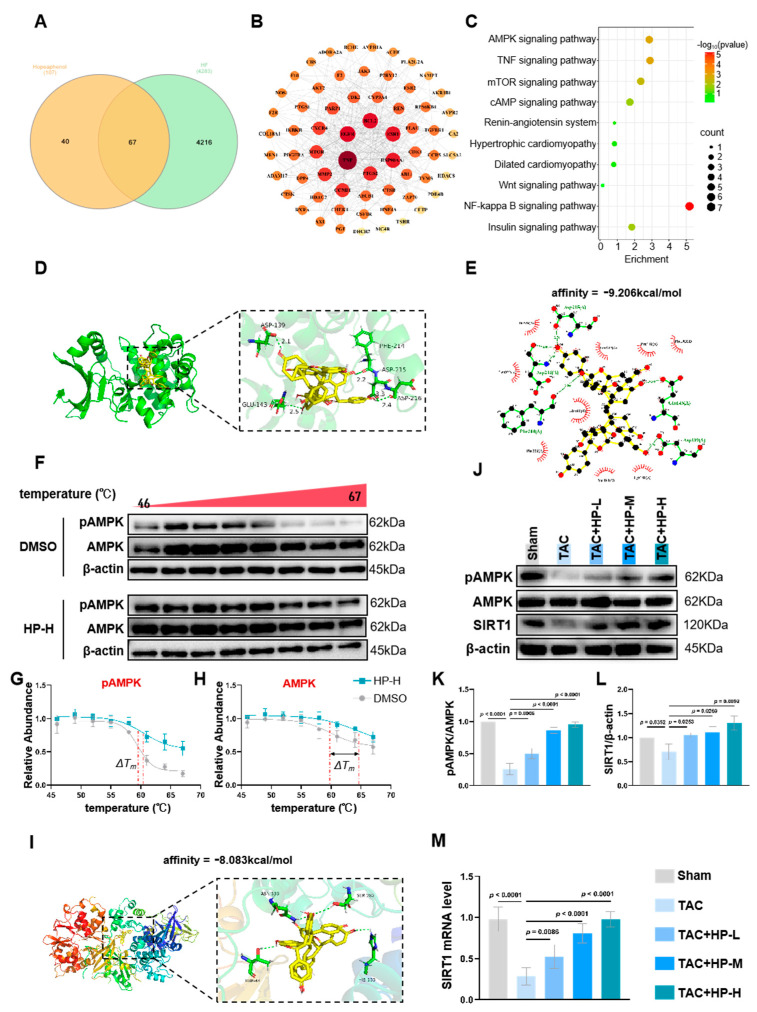
Hopeaphenol targets AMPK for cardiac protection. (**A**) Venn diagram of 67 overlapping hopeaphenol–heart failure targets. (**B**) PPI network (node size = degree; red: top 10 targets). (**C**) KEGG enrichment: AMPK pathways (FDR < 0.05). (**D**–**E**) Molecular docking of hopeaphenol with the human AMPK α2 kinase domain (PDB ID: 2H6D, binding affinity: −9.206 kcal/mol). (**F**) Cellular Thermal Shift Assay (CETSA) Western blot analysis of pAMPK and total AMPK protein stability in HL-1 cells treated with DMSO or Hopeaphenol (HP, 10 μM) across a temperature gradient (46–69 °C). β-actin served as a loading control (*n* = 3). (**G**) Quantification of the pAMPK protein thermal stability from (**F**). Hopeaphenol treatment induced a stabilization shift (ΔTm = +0.91 °C). (**H**) Quantification of the total AMPK protein thermal stability from (**F**). Hopeaphenol treatment induced a highly significant stabilization (ΔTm = +4.79 °C, *p* < 0.05 vs. DMSO control), providing direct evidence of target engagement. (**I**) Molecular docking of hopeaphenol with the full-length active-state AMPK heterotrimer (PDB: 6B2E). (**J**–**L**) pAMPK/AMPK and SIRT1 Western blot (**J**), density (**K**,**L**). (**M**) qPCR analysis of Sirt1 mRNA expression in myocardial tissues. Exact *p*-values for all post hoc comparisons are indicated on the graphs. Data are presented as mean ± SEM (*n* = 6). HP, Hopeaphenol.

Network pharmacology analysis revealed that there are 67 common targets shared between hopeaphenol and heart failure, representing 62.61% of the total targets of hopeaphenol (Figure 4A). Protein–protein interaction network analysis demonstrated that these targets constitute a highly interconnected module, with an average connectivity of 13.156 and a density of 0.209. Within this network, key downstream effectors of AMPK, such as CCND1 (connectivity = 28), MTOR (connectivity = 26), and AKT2 (connectivity = 13), were identified as central components (Figure 4B). KEGG pathway enrichment analysis further corroborated the significant activation of the AMPK signaling pathway (*p* < 0.05), with 60% of the annotated molecules in this pathway represented within the network (Figure 4C). Molecular docking was performed using two AMPK structures: the human AMPK α2 kinase domain (PDB ID: 2H6D, predominant cardiac catalytic isoform) and the full-length active-state AMPK heterotrimer (PDB: 6B2E). Results showed hopeaphenol had strong binding affinity to the active site of AMPK α2 (PDB ID: 2H6D) with a binding energy of −9.206 kcal/mol, forming stable hydrogen bonds with key residues (GLU-143 and ASP-139) (Figure 4D,E). Notably, hopeaphenol exhibited weaker binding affinity to the full-length active-state AMPK (PDB: 6B2E, binding energy: −8.083 kcal/mol), indicating a preference for the inactive kinase state.

To verify the direct interaction between Hopeaphenol (HP) and AMPK, we performed a Cellular Thermal Shift Assay (CETSA) combined with Western blot analysis. First, we assessed the thermal stability of phospho-AMPK (pAMPK): pAMPK protein is susceptible to degradation within the temperature range of 46–69 °C; in the DMSO control group, pAMPK degradation initiated at 59.5 °C, while in the HP-treated group, pAMPK degradation was significantly attenuated at this temperature (Figure 4F). To rule out the possibility that the signal shift reflected only phospho-epitope stabilization, we further detected the thermal stability of total AMPK using an antibody independent of phosphorylation status. The results showed that Hopeaphenol treatment induced a highly significant increase in the thermal stability of total AMPK protein, with a ΔTm of +4.79 °C compared to the DMSO control (*p* < 0.05), while the ΔTm for pAMPK was +0.91 °C (Figure 4G,H). This differential stabilization pattern was consistent with molecular docking results, collectively confirming HP’s state-dependent binding preference for inactive AMPK. Western blot analysis revealed that in the TAC model, myocardial pAMPK levels were significantly reduced while total AMPK expression remained unchanged; the AMPK downstream effector SIRT1 was also downregulated (Figure 4J–L). Notably, hopeaphenol treatment reversed these alterations in a dose-dependent manner, with pAMPK and SIRT1 levels increasing with rising HP concentrations (Figure 4J–L).

These findings demonstrate that hopeaphenol directly interacts with AMPK (preferentially the inactive state), thereby enhancing its stability, promoting its phosphorylation, and potentially activating the AMPK/SIRT1 signaling pathway (Figure 4).

### 3.5. Hopeaphenol Mitigates Ang II-Induced Cardiomyocyte Hypertrophy Via Activation of the AMPK/SIRT1 Pathway

**Figure 5:**
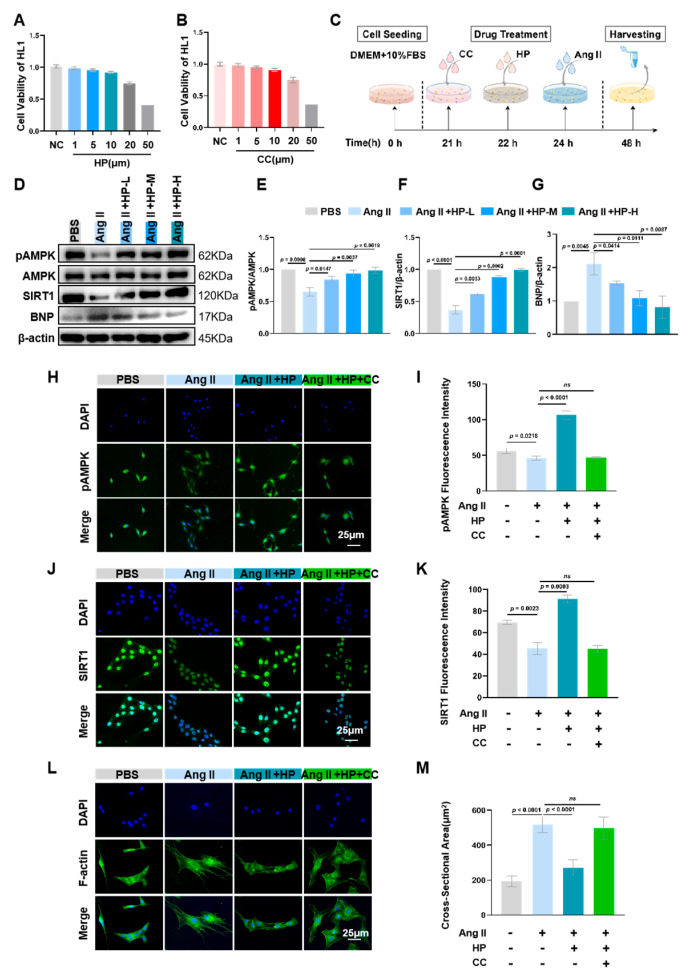
Hopeaphenol alleviates Ang II-induced cardiomyocyte hypertrophy Via AMPK/SIRT1 activation. (**A**,**B**) CCK-8 assay for non-toxic ranges of hopeaphenol (0–50 μM) and the AMPK inhibitor CC (0–50 μM) (24 h). (**C**) Cells modeling flowchart. (**D**–**G**) Western blot analysis of p-AMPK/AMPK (**E**), SIRT1 (**F**) and BNP (**G**) in HL-1 cells with densitometric analysis. (**H**–**K**) Immunofluorescence of p-AMPK (**H**) and SIRT1 (**J**) with DAPI (blue) (scale bar, 25 μm); quantification of fluorescence intensity (**I**,**K**). (**L**–**M**) Phalloidin staining (green) of F-actin with DAPI (blue) (**L**) and quantification of cell cross-sectional area (**M**) (scale bar, 25 μm). Cells were pretreated with HP (1, 5, 10 μM) for 2 h or CC (10 μM) for 1 h, followed by stimulation with Ang II (1 μM) for 24 h. Exact *p*-values for all post hoc comparisons are indicated on the graphs. Data are presented as mean ± SEM (*n* = 6). ns, not significant; HP, Hopeaphenol.

The CCK-8 assay was employed to ascertain the safe concentration range of hopeaphenol (1–10 μM), within which cell viability did not significantly differ from that of the normal control group (Figure 5A,B). Based on these findings, three concentrations—1 μM (low), 5 μM (medium), and 10 μM (high)—were selected for subsequent mechanistic investigations. Western blot analysis revealed that Ang II treatment markedly suppressed the expression of pAMPK and SIRT1 proteins in cardiomyocytes, which was accompanied by an upregulation of the cardiac hypertrophy marker BNP (Figure 5D–G). Treatment with hopeaphenol reversed these alterations in a dose-dependent manner, with expression levels of pAMPK and SIRT1 in the 10 μM hopeaphenol treatment group being 3.68-fold and 1.84-fold higher, respectively, compared to the Ang II group. Cellular immunofluorescence demonstrated that hopeaphenol treatment alone significantly enhanced the fluorescence signals of pAMPK and SIRT1. However, the concurrent application of the AMPK inhibitor Compound C completely abrogated these effects of hopeaphenol, resulting in pAMPK and SIRT1 expression levels reverting to those observed in the Ang II group (Figure 5H–K). Furthermore, phalloidin staining demonstrated that hopeaphenol treatment markedly decreased the surface area of cardiomyocytes, and this anti-hypertrophic effect was entirely negated by the presence of Compound C (Figure 5L–M). These findings indicate that the cardioprotective effect of hopeaphenol is contingent upon the activation of the AMPK/SIRT1 signaling pathway (Figure 5).

### 3.6. Hopeaphenol Mitigates Ang II-Induced Mitochondrial Dysfunction in HL-1 Cardiomyocytes Via the AMPK Pathway

**Figure 6:**
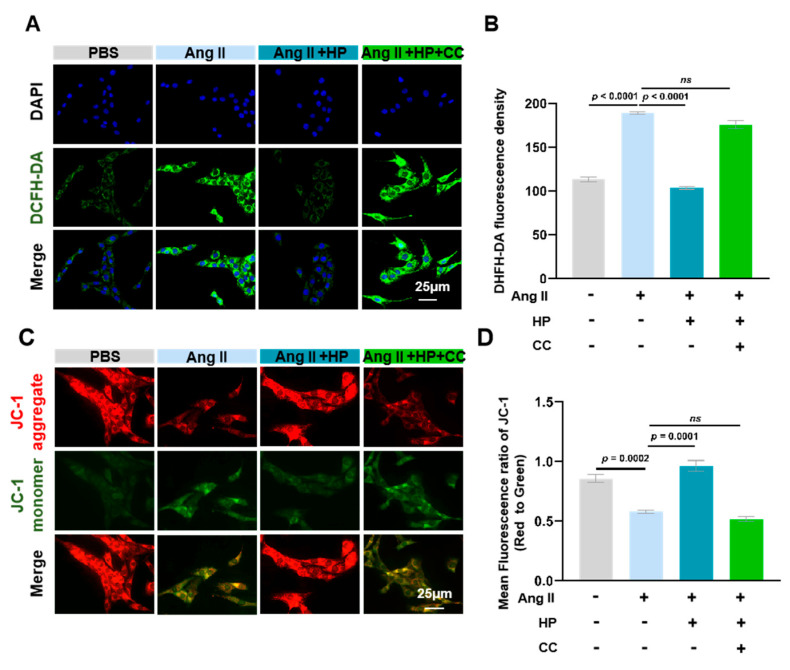
Hopeaphenol reduces mitochondrial ROS and enhances membrane potential in Ang II-treated HL-1 cardiomyocytes. (**A**,**B**) Mitochondrial ROS detection (DCFH-DA, green) (**A**) and quantification of fluorescence intensity (**B**) (scale bar = 25 μm). (**C**,**D**) JC-1 staining (red: J-aggregates, green: J-monomers) (**C**) and red/green fluorescence ratio (ΔΨm) (**D**) (scale bar = 25 μm). Cells were pretreated as in Figure 5. Exact *p*-values for all post hoc comparisons are indicated on the graphs. Data are presented as mean ± SEM (*n* = 6). ns, not significant; HP, Hopeaphenol.

Stimulation with Ang II markedly elevated mitochondrial reactive oxygen species (ROS) levels in HL-1 cardiomyocytes (Figure 6A,B) and led to a reduction in mitochondrial membrane potential (Figure 6C,D). Treatment with hopeaphenol significantly counteracted these aberrant alterations by decreasing mitochondrial ROS levels and restoring membrane potential. Notably, pre-treatment with the AMPK-specific inhibitor, Compound C, entirely abrogated the beneficial effects of hopeaphenol on both mitochondrial ROS levels and membrane potential. These findings substantiate that hopeaphenol ameliorates Ang II-induced mitochondrial oxidative stress and functional impairment through the activation of the AMPK pathway (Figure 6).

### 3.7. Inhibition of AMPK Activity Partially Blocks the Protective Effects of Hopeaphenol on Cardiomyocytes

**Figure 7:**
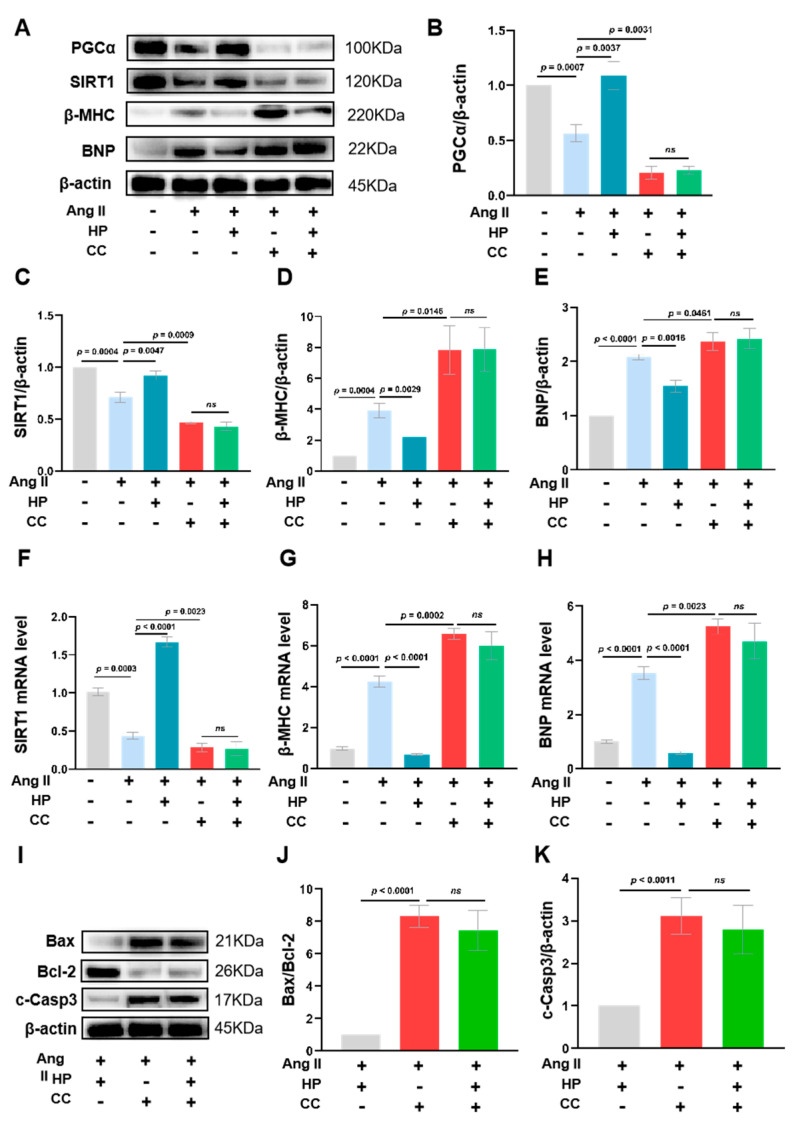
AMPK inhibition by Compound C abolishes Hopeaphenol-mediated protection against Ang II-induced hypertrophy and apoptosis in HL-1 cardiomyocytes. (**A**–**E**) Western blot analysis of PGC-1α, SIRT1, β-MHC, and BNP (**A**) and their quantification ((**B**–**E**), normalized to β-actin). (**F**–**H**) qPCR analysis of SIRT1, BNP, and β-MHC mRNA. (**I**–**K**) Western blot analysis of Bax, Bcl-2, and cleaved caspase-3 (**I**), and quantification of the Bax/Bcl-2 ratio (**J**) and cleaved caspase-3 (**K**). Cells were pretreated as in Figure 5. Exact *p* values for all post hoc comparisons are indicated on the graphs. Data are presented as mean ± SEM (*n* = 3 for panels (**I**–**K**); *n* = 6 for others). ns, not significant; c-Casp3, cleaved caspase-3; HP, Hopeaphenol.

Western blot analyses revealed that Angiotensin II (Ang II) treatment markedly suppressed the protein expression of AMPK downstream effectors, PGC-1α and SIRT1, in HL-1 cardiomyocytes. Concurrently, Ang II treatment led to an upregulation of cardiac hypertrophy markers, BNP and β-MHC. Intervention with hopeaphenol significantly ameliorated these aberrant changes. However, pre-treatment with Compound C entirely negated the effects of hopeaphenol (Figure 7A–E). Further, qPCR analysis corroborated that Ang II significantly reduced SIRT1 mRNA levels while elevating the mRNA expression of BNP and β-MHC. Hopeaphenol treatment substantially improved these parameters, but pre-treatment with Compound C completely nullified hopeaphenol’s protective effects (Figure 7F–H). Additionally, hopeaphenol intervention significantly mitigated the Ang II-induced increase in the BAX/BCL2 ratio and the elevation of Cleaved caspase-3 protein levels. This anti-apoptotic effect of hopeaphenol was entirely obstructed by pre-treatment with Compound C (Figure 7I–K). The aforementioned findings suggest that hopeaphenol exerts protective effects against cardiac hypertrophy and apoptosis primarily by activating the AMPK/SIRT1 signaling pathway, though the involvement of other potential pathways cannot be completely excluded due to the off-target effects of Compound C. Notably, the inhibition of AMPK activity substantially abrogates the cardioprotective effects of hopeaphenol, which strongly supports the critical role of AMPK in mediating these beneficial effects (Figure 7).

## 4. Discussion

This study demonstrates that hopeaphenol alleviates pressure overload-induced cardiac hypertrophy and delays heart failure progression by activating the AMPK signaling pathway (with direct binding supported by CETSA and docking), which enhances mitochondrial energy metabolism and reduces oxidative stress. Below, we elaborate on the pathological mechanisms underlying cardiac hypertrophy and the specific role of hopeaphenol in modulating these processes.

Cardiac hypertrophy, a pathological remodeling response to overload, is characterized by a vicious cycle involving abnormal mitochondrial energy metabolism and oxidative stress [3,23,24]. This pathological link is further supported by clinical research in human hypertrophic cardiomyopathy: studies have shown mitochondrial dysfunction is closely associated with structural disruptions in cardiomyocytes, and this dysfunction can be partially reversed by enhancing NADH-driven mitochondrial respiration—highlighting the therapeutic potential of targeting mitochondrial metabolism in hypertrophy [25]. It is initiated by cardiomyocytes sensing and responding to mechanical load, in which mechanical stimuli are converted into biochemical signals Via mechanotransduction. In the early stages, adaptive changes in myocardial structure and function can maintain cardiac pumping efficiency [24]; however, sustained or excessive load triggers maladaptive remodeling, disrupting cardiomyocyte metabolic homeostasis. As a highly energy-dependent organ, the heart relies on continuous ATP synthesis by mitochondria—ATP reserves support only limited contractions and must be replenished Via mitochondrial oxidative phosphorylation. Mitochondria also regulate contractile function and cell viability, and mitochondrial dysfunction is a hallmark of heart failure [26,27,28,29]. Prolonged pressure overload, such as in the transverse aortic constriction (TAC) model used here, impairs mitochondrial core functions: it disrupts the respiratory chain, increases electron leakage during electron transfer, and induces excessive reactive oxygen species (ROS) production [7,24]. Excessive ROS further exacerbates mitochondrial dysfunction (protein misfolding), damages cardiomyocyte structures (membranes, organelles), and activates pro-apoptotic and pro-fibrotic pathways, forming a “vicious cycle” of mitochondrial impairment, ROS accumulation, and aggravated myocardial damage [24,29,30].

In this study, TAC mice exhibited reduced left ventricular ejection fraction (LVEF) and fractional shortening (LVFS), enlarged ventricular cavities (increased LVIDd and LVIDs), cardiomyocyte hypertrophy (HE and WGA staining), fibrosis (Masson staining), and systemic oxidative stress/metabolic disturbances (decreased serum SOD activity and ATP levels, increased serum MDA levels). Hopeaphenol treatment dose-dependently ameliorated these changes; notably, in vitro experiments confirmed HP directly improved cardiac mitochondrial function by reducing mitochondrial ROS and restoring membrane potential. These results confirm that hopeaphenol disrupts the “mitochondrial dysfunction–oxidative stress–cardiac hypertrophy” vicious cycle by improving systemic energy metabolism and augmenting antioxidant capacity.

Hopeaphenol’s protective effects are closely linked to AMPK activation. AMPK, a central cellular energy sensor activated by increased AMP/ATP ratios, maintains mitochondrial homeostasis by regulating downstream targets [11,31,32]. Molecular docking confirmed that hopeaphenol interacts directly with AMPK by forming stable bonds with key active-site residues, supporting a specific molecular association between the two. CETSA experiments further validated this direct interaction, showing that hopeaphenol enhances pAMPK’s thermal stability and reduces its degradation—corroborating the docking results and reinforcing that hopeaphenol binds AMPK to modulate its function. Functionally, hopeaphenol upregulated AMPK and SIRT1 expression in TAC mouse myocardium; SIRT1, a downstream AMPK target, promotes mitochondrial biogenesis and antioxidant function Via deacetylation of PGC-1α [33]. Notably, in vitro, hopeaphenol’s beneficial effects on Ang II-induced HL-1 cardiomyocyte hypertrophy (reduced cell area, downregulated BNP) and mitochondrial dysfunction (decreased ROS, restored membrane potential) were abolished by the AMPK inhibitor Compound C, confirming AMPK as a critical mediator of hopeaphenol’s effects.

Building on these experimental findings (direct binding Via docking/CETSA, functional upregulation of AMPK/SIRT1, and dependence on AMPK activity), we propose a model where hopeaphenol’s direct binding to inactive AMPK (supported by docking and CETSA), combined with the well-established mechanism that LKB1 serves as a classical upstream phosphorylating kinase of AMPK [34], is speculated to sensitize the kinase to phosphorylation by upstream regulators like LKB1. The activation of AMPK occurs through the phosphorylation of T172, a process controlled by LKB1, and the enhanced LKB1/AMPK pathway stimulates the TSC1/TSC2 complex, subsequently suppressing mTOR activity [34]. This priming effect leads to enhanced AMPK phosphorylation (pAMPK upregulation) and subsequent activation of downstream effectors (SIRT1, PGC-1α). SIRT1 then participates in a positive feedback loop—by deacetylating PGC-1α, it promotes mitochondrial biogenesis and antioxidant function, amplifying AMPK-mediated metabolic improvements. This model explains how HP’s binding initiates AMPK activation without requiring it to be a direct orthosteric activator, and highlights the potential role of upstream kinases in mediating the full signaling cascade.

This proposed model integrates our key experimental observations. These include direct hopeaphenol-AMPK binding (docking/CETSA), elevated pAMPK (WB) and SIRT1 upregulation. It also bridges a knowledge gap. It links the earlier observed hopeaphenol-AMPK binding (and subsequent stability enhancement) to the functional increase in pAMPK—resolving how physical interaction translates to pathway activation—a marker of AMPK activation and subsequent downstream activation. This link relies on speculated LKB1-mediated phosphorylation sensitization. Direct kinase assays were not performed. Even so, this framework prepares for comparing hopeaphenol with other AMPK activators like resveratrol. It highlights how hopeaphenol’s unique mechanism connects to its structural and functional advantages.

While our study delineates the central role of the AMPK/SIRT1 axis, our KEGG enrichment analysis (Figure 4C) also suggests the potential involvement of other downstream pathways such as mTOR signaling and autophagy—key regulators of cardiac hypertrophy. It has been shown that the activation of AMPK/mTOR signaling is linked to the activation of autophagy [35]. Given established crosstalk between AMPK activation, mTOR inhibition (which suppresses pathological protein synthesis), and enhanced autophagic flux (which clears damaged mitochondria), these pathways may contribute to hopeaphenol’s comprehensive cardioprotective effects. Due to time and scope constraints, we did not experimentally validate these pathways in the current study; future investigations will be essential to determine their specific contributions and whether they act in synergy with AMPK/SIRT1 to amplify therapeutic benefits. This potential multi-pathway regulatory feature aligns with hopeaphenol’s structural basis for multi-target activity (foreshadowed in the model’s emphasis on “unique mechanism”), as its multimeric structure enables interactions with multiple targets—laying the groundwork for the following discussion of structural advantages.

As a resveratrol tetramer, hopeaphenol exhibits structural and functional advantages over its monomeric parent, building on foundational work by Seya et al. [18]. Their study identified that resveratrol tetramers display divergent bioactivities: vitisin A induces cardiomyocyte apoptosis, while hopeaphenol suppresses it—underscoring that stilbene oligomer function depends on specific stereochemistry rather than just monomer composition. Our findings extend this work by demonstrating that hopeaphenol’s anti-apoptotic phenotype translates to anti-hypertrophic effects Via AMPK/SIRT1 activation, providing a mechanistic explanation for its protective role first observed by Seya et al. Stilbene oligomers, including hopeaphenol, have improved biological properties: their multimeric structure enhances stability and resistance to metabolic degradation, ensuring higher bioavailability compared to resveratrol monomers. This structural feature also confers multi-target activity—hopeaphenol not only directly binds to AMPK but also interacts more effectively with molecules such as angiotensin-converting enzyme (ACE) than resveratrol [36], thereby amplifying its protective effects [18]. In contrast, resveratrol has limitations: its effects are dose-dependent and biphasic (antioxidant at low doses, pro-oxidant at ≥50 μM, causing DNA damage and mitochondrial dysfunction in endothelial cells [37,38,39,40]), with toxicity to normal cells (rat thymocytes and fibroblasts [41,42]) and potential drug–drug interactions via CYP3A4 inhibition [43,44]. In clinical settings, administering high doses (2–5 g/day) of certain compounds is often associated with gastrointestinal discomfort (such as diarrhea and nausea), abnormal liver function, and potential nephrotoxicity [45,46]. Nevertheless, our results confirm hopeaphenol’s low toxicity: Hopeaphenol ameliorates cardiac hypertrophy in a dose-dependent manner at 5–20 mg/kg (in vivo) or 1–10 μM (in vitro) without significant toxicity. More importantly, hopeaphenol mediates the activation of the AMPK/SIRT1 pathway through direct binding to AMPK—this interaction is supported by comprehensive structural and functional evidence: molecular docking with two AMPK structures (cardiac-predominant α2 kinase domain, PDB 2H6D; full-length active heterotrimer, PDB 6B2E) shows hopeaphenol binds the inactive α2 isoform with higher affinity (binding energy: −9.206 kcal/mol vs. −8.083 kcal/mol for 6B2E) Via stable hydrogen bonds with GLU-143 and ASP-139; meanwhile, Cellular Thermal Shift Assay (CETSA) confirms this direct binding by significantly enhancing total AMPK thermal stability (ΔTm: +4.79 °C) compared to the DMSO control. This state-dependent, target-specific mechanism is clearer and more reliable than resveratrol, which acts independently of the AMPK pathway at low concentrations.

In a clinical context, this study identifies hopeaphenol as a promising therapeutic candidate for cardiac hypertrophy, emphasizing the significance of the AMPK/SIRT1 signaling pathway in ameliorating myocardial metabolic disorders. Given that aberrant energy metabolism is an early indicator in the progression from hypertrophy to heart failure [6], hopeaphenol’s capacity to enhance cardiac function and mitigate remodeling in TAC mice indicates its potential utility for early intervention in pressure overload-induced hypertrophy (hypertension, aortic valve stenosis). However, several limitations must be addressed: (1) the study’s reliance on the TAC model necessitates validation across other etiologies of hypertrophy, such as volume overload from mitral regurgitation or myocardial infarction; (2) the investigation into upstream AMPK regulators, such as LKB1, and the complete activation cascade remains incomplete; (3) Elucidating the roles of specific AMPKα subunits through genetic approaches (knockout mouse models) to confirm AMPK’s essential role; (4) Developing nano-delivery systems to improve bioavailability, addressing a common challenge in translating natural polyphenols to clinical use. It is important to note that the use of Compound C, a pharmacological AMPK inhibitor, has limitations due to its potential off-target effects. While our data consistently show that Compound C blocks the beneficial effects of hopeaphenol, which strongly suggests the involvement of AMPK, this evidence alone cannot conclusively prove that the effects are exclusively mediated through AMPK. To more definitively establish the essential role of AMPK, future studies employing genetic approaches, such as siRNA-mediated knockdown or CRISPR/Cas9-mediated knockout of AMPKα in cardiomyocytes, are warranted. These methods would provide higher specificity and strengthen the conclusion that hopeaphenol’s cardioprotective effects are primarily dependent on AMPK signaling.

From a translational perspective, currently, hopeaphenol holds promise as a candidate for future therapeutic development rather than an immediately translatable clinical agent. Notwithstanding the promising cardioprotective effects observed, the clinical translational potential of hopeaphenol remains to be fully established—particularly addressing the limitations of pharmacological inhibitors (Compound C) noted earlier. Future investigations are therefore imperative, with key directions including: (1) Characterizing its pharmacokinetic profile, tissue distribution, and chronic toxicity in advanced animal models; (2) Evaluating its effects on human cardiomyocyte organoids or other human-relevant systems to bridge the gap between preclinical findings and potential clinical application; (3) Elucidating the roles of specific AMPKα subunits through genetic approaches (knockout mouse models) to confirm AMPK’s essential role; (4) Developing nano-delivery systems to improve bioavailability, addressing a common challenge in translating natural polyphenols to clinical use. These future directions will further validate the mechanisms underlying hopeaphenol’s action.

## 5. Conclusions

In this study, integrated in vivo (TAC mouse model), in vitro (Ang II-induced HL-1 cardiomyocytes), and network pharmacology experiments confirm that hopeaphenol exerts a protective effect against pressure overload-induced cardiac hypertrophy and delays heart failure progression.Mechanistically, hopeaphenol directly binds to AMPK (validated by molecular docking with two AMPK structures and CETSA) to activate the AMPK/SIRT1 signaling pathway. This activation improves mitochondrial energy metabolism, reduces oxidative stress, mitigates myocardial hypertrophy and fibrosis, and ultimately restores cardiac function—effects that are abolished by the AMPK inhibitor Compound C, confirming AMPK as a critical mediator.Compared with its monomer resveratrol, hopeaphenol has structural and functional advantages: higher bioavailability (due to enhanced metabolic stability), lower toxicity (effective at 5–20 mg/kg in vivo or 1–10 μM in vitro without significant cytotoxicity), and a clearer, more target-specific mechanism (state-dependent binding to inactive AMPK, avoiding resveratrol’s biphasic effects and off-target risks).

**Figure 8:**
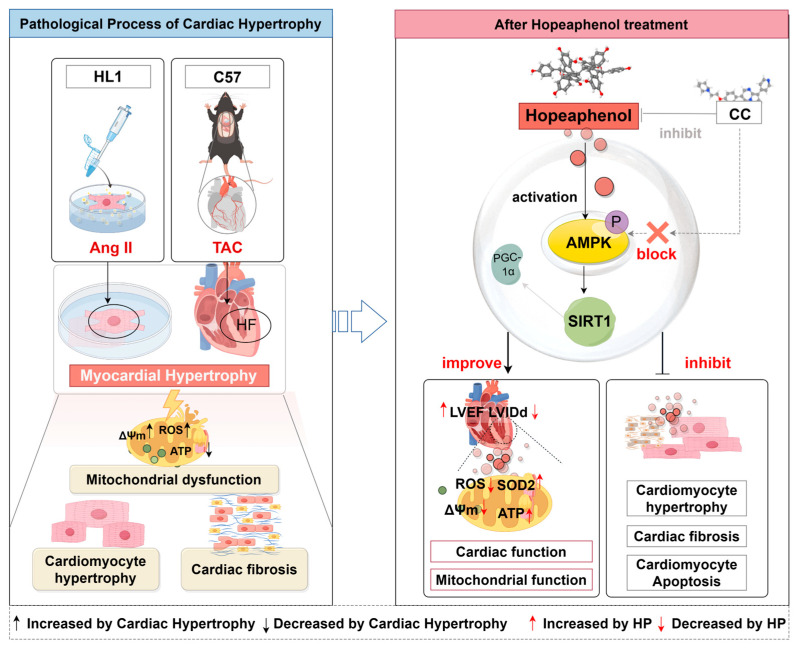
Schematic of hopeaphenol alleviating cardiac hypertrophy. The left panel shows the Ang II/TAC-induced pathological process (HL-1 cells, C57 mice), characterized by mitochondrial dysfunction, cardiomyocyte hypertrophy, and fibrosis. The right panel depicts that Hopeaphenol primarily activates the AMPK/SIRT1 signaling pathway to improve cardiac and mitochondrial function, and to inhibit hypertrophy, fibrosis, and apoptosis; notably, due to the potential off-target effects of CC (Compound C), the involvement of other pathways in these processes cannot be completely ruled out. Meanwhile, CC (Compound C) blocks the activity of AMPK, which in turn abrogates the aforementioned beneficial effects of Hopeaphenol—this observation strongly supports the role of AMPK in mediating Hopeaphenol’s cardioprotection (by Figdraw). CC, Compound C.

Collectively, these findings identify hopeaphenol as a promising candidate for the prevention and treatment of cardiac hypertrophy-related heart failure, while also highlighting the need for further research: validating its efficacy in diverse hypertrophy models, clarifying the role of specific AMPKα subunits via genetic approaches, and optimizing nano-delivery systems to improve its clinical translational potential (Figure 8).

## Figures and Tables

**Table 1:** qPCR Primer Sequences.

Gene	Forward Primer (5′–3′)	Reverse Primer (5′–3′)
AMPK	GTCAAAGCCGACCCAATGATA	CGTACACGCAAATAATAGGGGTT
SIRT1	TGATTGGCACCGATCCTCG	CCACAGCGTCATATCATCCAG
β-MHC	CCTGCGGAAGTCTGAGAAGG	CTCGGGACACGATCTTGGC
BNP	GAGGTCACTCCTATCCTCTGG	GAGGTCACTCCTATCCTCTGG
GAPDH	AGGTCGGTGTGAACGGATTTG	GGGGTCGTTGATGGCAACA

## Data Availability

The raw data supporting the conclusions of this article will be made available by the authors without undue reservation.

## Abbreviations

AMPK: AMP-activated protein kinase; ATP, adenosine triphosphate; ADP, adenosine diphosphate; AngII, angiotensin II; AKT2, protein kinase B β; ACE, angiotensin-converting enzyme; AMPKα, AMP-activated protein kinase α; BNP, brain natriuretic peptide; BAX, BCL2-associated X protein; BCL2, B-cell lymphoma 2; CC, compound C; FBS, fetal bovine serum; CYP3A4, cytochrome P450 3A4; cCasp3, cleaved caspase-3; CCND1, cyclin D1; EF, ejection fraction; F-actin, filamentous actin; FS, fractional shortening; HP, Hopeaphenol; HP-L, Hopeaphenol low; HP-M, Hopeaphenol middle; HP-H, Hopeaphenol high; HE, hematoxylin and eosin; LVIDd, left ventricular internal dimension at end-diastole; LVIDs, left ventricular internal dimension at end-systole; LVPWd, left ventricular posterior wall thickness at end-diastole; LVPWs, left ventricular posterior wall thickness at end-systole; LV Mass, left ventricular mass; LKB1, liver kinase B1; MET, metformin; MDA, malondialdehyde; mTOR, mammalian target of rapamycin; pAMPK, phosphorylated AMP-activated protein kinase; PGC-1α, peroxisome proliferator-activated receptor gamma coactivator 1-alpha; ROS, reactive oxygen species; SIRT1, silent information regulator 1; SOD, superoxide dismutase; TAC, transverse aortic constriction; WGA, wheat germ agglutinin; β-MHC, β-myosin heavy chain.

## References

[1] Vásquez-Trincado C., García-Carvajal I., Pennanen C., Parra V., Hill J.A., Rothermel B.A., Lavandero S. (2016). Mitochondrial dynamics, mitophagy and cardiovascular disease. J. Physiol..

[2] Ye B., Zhou H., Chen Y., Luo W., Lin W., Zhao Y., Han J., Han X., Huang W., Wu G. (2023). USP25 Ameliorates Pathological Cardiac Hypertrophy by Stabilizing SERCA2a in Cardiomyocytes. Circ. Res..

[3] Nakamura M., Sadoshima J. (2018). Mechanisms of physiological and pathological cardiac hypertrophy Nature reviews. Cardiology.

[4] Barry S.P., Davidson S.M., Townsend P.A. (2008). Molecular regulation of cardiac hypertrophy. Int. J. Biochem. Cell Biol..

[5] Lopaschuk G.D., Jaswal J.S. (2010). Energy metabolic phenotype of the cardiomyocyte during development, differentiation, and postnatal maturation. J. Cardiovasc. Pharmacol..

[6] Zhang L., Ussher J.R., Sankaralingam S., Wagg C., Zaugg M., Lopaschuk G.D. (2013). Cardiac insulin-resistance and decreased mitochondrial energy production precede the development of systolic heart failure after pressure-overload hypertrophy. Circ. Heart Fail..

[7] Gao F., Liang T., Lu Y.W., Fu X., Dong X., Pu L., Hong T., Zhou Y., Zhang Y., Liu N. (2023). A defect in mitochondrial protein translation influences mitonuclear communication in the heart. Nat. Commun..

[8] Ritterhoff J.T.R. (2023). Metabolic mechanisms in physiological and pathological cardiac hypertrophy: New paradigms and challenges. Nat. Rev. Cardiol..

[9] Doenst T., Nguyen T.D., Abel E.D. (2013). Cardiac metabolism in heart failure: Implications beyond ATP production. Circ. Res..

[10] Peng S., Lu X.F., Qi Y.D., Li J., Xu J., Yuan T.Y., Wu X.Y., Ding Y., Li W.H., Zhou G.-Q. (2020). LCZ696 Ameliorates Oxidative Stress and Pressure Overload-Induced Pathological Cardiac Remodeling by Regulating the Sirt3/MnSOD Pathway. Oxidative Med. Cell. Longev..

[11] O’brien C.M., Mulukutla B.C., Mashek D.G., Hu W.-S. (2020). Regulation of Metabolic Homeostasis in Cell Culture Bioprocesses. Trends Biotechnol..

[12] Herzig S., Shaw R.J. (2018). AMPK: Guardian of metabolism and mitochondrial homeostasis. Nat. Rev. Mol. Cell Biol..

[13] Zhu C., Wang M., Yu X., Shui X., Tang L., Chen Z., Xiong Z. (2022). lncRNA NBR2 attenuates angiotensin II-induced myocardial hypertrophy through repressing ER stress via activating LKB1/AMPK/Sirt1 pathway. Bioengineered.

[14] Dolinsky V.W.S.C., Rogan K.J., Chan A.Y., Nagendran J., Wang S., Dyck J.R.B. (2015). Resveratrol prevents pathological but not physiological cardiac hypertrophy. J. Mol. Med..

[15] Dolinsky V.W., Chakrabarti S., Pereira T.J., Oka T., Levasseur J., Beker D., Zordoky B.N., Morton J.S., Nagendran J., Lopaschuk G.D. (2013). Resveratrol prevents hypertension and cardiac hypertrophy in hypertensive rats and mice. Biochim. Biophys. Acta.

[16] Giuliani C., Iezzi M., Ciolli L., Hysi A., Bucci I., Di Santo S., Rossi C., Zucchelli M., Napolitano G. (2017). Resveratrol has anti-thyroid effects both in vitro and in vivo. Food Chem. Toxicol. Int. J. Publ. Br. Ind. Biol. Res. Assoc..

[17] Shaito A., Posadino A.M., Younes N., Hasan H., Halabi S., Alhababi D., Al-Mohannadi A., Abdel-Rahman W.M., Eid A.H., Nasrallah G.K. (2020). Potential Adverse Effects of Resveratrol: A Literature Review. Int. J. Mol. Sci..

[18] Seya K., Kanemaru K., Sugimoto C., Suzuki M., Takeo T., Motomura S., Kitahara H., Niwa M., Oshima Y., Furukawa K.-I. (2009). Opposite effects of two resveratrol (trans-3,5,4′-trihydroxystilbene) tetramers, vitisin A and hopeaphenol, on apoptosis of myocytes isolated from adult rat heart. J. Pharmacol. Exp. Ther..

[19] Dealmeida A.C., Van Oort R.J., Wehrens X.H. (2010). Transverse aortic constriction in mice. J. Vis. Exp. JoVE.

[20] Fu Y.N., Xiao H., Ma X.W., Jiang S.-Y., Xu M., Zhang Y.-Y. (2011). Metformin attenuates pressure overload-induced cardiac hypertrophy via AMPK activation. Acta Pharmacol. Sin..

[21] Hwangbo D.S., Lee H.Y., Abozaid L.S., Min K.-J. (2020). Mechanisms of Lifespan Regulation by Calorie Restriction and Intermittent Fasting in Model Organisms. Nutrients.

[22] Xiao Z., Dai C., Yu T., Zhu J., Pan Y., Shuai W., Kong B., Huang H. (2024). Ubiquitin specific protease 38 aggravates pathological cardiac remodeling by stabilizing phospho-TBK1. Int. J. Biol. Sci..

[23] Shimizu I., Minamino T. (2016). Physiological and pathological cardiac hypertrophy. J. Mol. Cell. Cardiol..

[24] Gallo G., Rubattu S., Volpe M. (2024). Mitochondrial Dysfunction in Heart Failure: From Pathophysiological Mechanisms to Therapeutic Opportunities. Int. J. Mol. Sci..

[25] Nollet E.E., Duursma I., Rozenbaum A., Eggelbusch M., I Wüst R.C., Schoonvelde S.A.C., Michels M., Jansen M., van der Wel N.N., Bedi K.C. (2023). Mitochondrial dysfunction in human hypertrophic cardiomyopathy is linked to cardiomyocyte architecture disruption and corrected by improving NADH-driven mitochondrial respiration. Eur. Heart J..

[26] Lyon R.C., Zanella F., Omens J.H., Sheikh F. (2015). Mechanotransduction in cardiac hypertrophy and failure. Circ. Res..

[27] Ingwall J.S. (2009). Energy metabolism in heart failure and remodelling. Cardiovasc. Res..

[28] Dai D.F., Hsieh E.J., Liu Y., Chen T., Beyer R.P., Chin M.T., MacCoss M.J., Rabinovitch P.S. (2012). Mitochondrial proteome remodelling in pressure overload-induced heart failure: The role of mitochondrial oxidative stress. Cardiovasc. Res..

[29] Smyrnias I., Gray S.P., Okonko D.O., Sawyer G., Zoccarato A., Catibog N., López B., González A., Ravassa S., Díez J. (2019). Cardioprotective Effect of the Mitochondrial Unfolded Protein Response During Chronic Pressure Overload. J. Am. Coll. Cardiol..

[30] Goh K.Y., He L., Song J., Jinno M., Rogers A.J., Sethu P., Halade G.V., Rajasekaran N.S., Liu X., Prabhu S.D. (2019). Mitoquinone ameliorates pressure overload-induced cardiac fibrosis and left ventricular dysfunction in mice. Redox Biol..

[31] Feng Y., Zhang Y., Xiao H. (2018). AMPK and cardiac remodelling. Sci. China Life Sci..

[32] Dong H.W., Zhang L.F., Bao S.L. (2018). AMPK regulates energy metabolism through the SIRT1 signaling pathway to improve myocardial hypertrophy. Eur. Rev. Med. Pharmacol. Sci..

[33] Tang B.L. (2016). Sirt1 and the Mitochondria. Mol. Cells.

[34] Su P.-S., Doerksen R.J., Chen S.-H., Sung W.-C., Juan C.-C., Rawendra R.D., Chen C.-R., Li J.-W., Aisha, Huang T.-C. (2015). Screening and profiling stilbene-type natural products with angiotensin-converting enzyme inhibitory activity from *Ampelopsis brevipedunculata* var. hancei (Planch.) Rehder. J. Pharm. Biomed. Anal..

[35] Häusl A.S., Bajaj T., Brix L.M., Pöhlmann M.L., Hafner K., De Angelis M., Nagler J., Dethloff F., Balsevich G., Schramm K.-W. (2022). Mediobasal hypothalamic FKBP51 acts as a molecular switch linking autophagy to whole-body metabolism. Sci. Adv..

[36] Liu W., Zhao Y., Wang G., Feng S., Ge X., Ye W., Wang Z., Zhu Y., Cai W., Bai J. (2022). TRIM22 inhibits osteosarcoma progression through destabilizing NRF2 and thus activation of ROS/AMPK/mTOR/autophagy signaling. Redox Biol..

[37] Posadino A.M., Cossu A., Giordo R., Zinellu A., Sotgia S., Vardeu A., Hoa P.T., Van Nguyen L.H., Carru C., Pintus G. (2015). Resveratrol alters human endothelial cells redox state and causes mitochondrial-dependent cell death. Food Chem. Toxicol..

[38] Calabrese E.J., Mattson M.P., Calabrese V. (2010). Resveratrol commonly displays hormesis: Occurrence and biomedical significance. Hum. Exp. Toxicol..

[39] Hadi S.M., Ullah M.F., Azmi A.S., Ahmad A., Shamim U., Zubair H., Khan H.Y. (2010). Resveratrol mobilizes endogenous copper in human peripheral lymphocytes leading to oxidative DNA breakage: A putative mechanism for chemoprevention of cancer. Pharm. Res..

[40] Fujimoto A., Sakanashi Y., Matsui H., Oyama T., Nishimura Y., Masuda T., Oyama Y. (2009). Cytometric analysis of cytotoxicity of polyphenols and related phenolics to rat thymocytes: Potent cytotoxicity of resveratrol to normal cells. Basic. Clin. Pharmacol. Toxicol..

[41] Berardi V., Ricci F., Castelli M., Galati G., Risuleo G. (2009). Resveratrol exhibits a strong cytotoxic activity in cultured cells and has an antiviral action against polyomavirus: Potential clinical use. J. Exp. Clin. Cancer Res..

[42] Deng R., Xu C., Chen X., Chen P., Wang Y., Zhou X., Jin J., Niu L., Ying M., Huang M. (2014). Resveratrol suppresses the inducible expression of CYP3A4 through the pregnane X receptor. J. Pharmacol. Sci..

[43] Detampel P.B.M., Krähenbühl S., Huwyler J. (2012). Drug interaction potential of resveratrol. Drug Metab. Rev..

[44] Atmaca N.Y.E., Güner B., Kabakçi R., Bilmen F.S. (2014). Effect of resveratrol on hematological and biochemical alterations in rats exposed to fluoride. BioMed Res. Int..

[45] Wang Y., Cui H., Niu F., Liu S.-L., Li Y., Zhang L.-M., Du H.-B., Zhao Z.-G., Niu C.-Y. (2018). Effect of Resveratrol on Blood Rheological Properties in LPS-Challenged Rats. Front. Physiol..

[46] Crowell J.A., Korytko P.J., Morrissey R.L., Booth T.D., Levine B.S. (2004). Resveratrol-associated renal toxicity. Toxicol. Sci..

